# The Physician’s Hand-Book for 1890

**Published:** 1890-02

**Authors:** 


**The Physician's Hand-Book for 1890.** By A. D. ELMER, M. D.

This popular standard hand-book, which has been issued for over thirty years by the W. A. Townsend Publishing Co., has been transferred to the publishing house of G. P. Putnam's Sons, 27 and 29

West 23d street, New York. This hand-book combines the conveniences of a diary with those of a manual, and is of great benefit to the practising physician. This edition has been thoroughly revised—containing all the recent discoveries in materia medica and therapeutics, together with the new remedies which of late have come in vogue.

It is bound in English morocco, pocket-book form, and is retailed at $1.50.

